# (Acetato-κ*O*)bis­(2,2′-bipyridyl-κ^2^ *N*,*N*′)copper(II)–ethyl sulfate–methyl sulfate (1/0.5/0.5)

**DOI:** 10.1107/S1600536808037331

**Published:** 2008-11-20

**Authors:** Zhi-Gang Wen, Mao-Lian Li

**Affiliations:** a Department of Chemistry and Chemical Engineering, Qiannan Normal College for Nationalities, Duyun, Guizhou 558000, People’s Republic of China; b Department of Chemistry and Biology, Qinzhou University, Qinzhou, Guangxi 535000, People’s Republic of China

## Abstract

In the title complex, [Cu(C_2_H_3_O_2_)(C_10_H_8_N_2_)_2_](CH_3_CH_2_OSO_3_)_0.5_(CH_3_OSO_3_)_0.5_, the Cu^II^ ion is bis-chelated by two 2,2′-bipyridine lignds and coordinated by an O atom of an acetate ligand in a CuN_4_O disorted square-pyramidal environment. In the structure, equal amounts of methyl sulfate and ethyl sulfate anions are disordered on the same crystallographic sites. The crystal structure is stabilized by weak inter­molecular C—H⋯O inter­actions.

## Related literature

For genernal background to supra­molecular assembly and crystal engineering, see: Aakeröy *et al. *(1998 ▶); Batten & Robson (1998 ▶); Yaghi *et al.* (1998 ▶); Kitagawa *et al.* (2004 ▶); Lu *et al.* (2006 ▶). For related strutures, see: Akrivos *et al.* (1994 ▶); Blake *et al.* (2000 ▶); Belokon *et al.* (2002 ▶); Lopez-Sandoval *et al.* (2004 ▶).
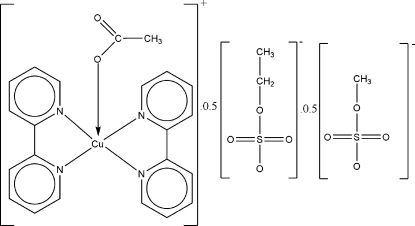


## Experimental

### 

#### Crystal data

[Cu(C_2_H_3_O_2_)(C_10_H_8_N_2_)_2_](C_2_H_5_O_4_S)_0.5_(CH_3_O_4_S)_0.5_
*M* *_r_* = 553.06
Triclinic, 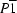

*a* = 7.1314 (7) Å
*b* = 13.1173 (13) Å
*c* = 13.2783 (14) Å
α = 91.875 (1)°
β = 104.673 (1)°
γ = 101.162 (1)°
*V* = 1174.5 (2) Å^3^
*Z* = 2
Mo *K*α radiation
μ = 1.07 mm^−1^
*T* = 291 (2) K
0.36 × 0.27 × 0.22 mm

#### Data collection

Bruker SMART CCD area-detector diffractometer
Absorption correction: multi-scan (*SADABS*; Sheldrick, 1996 ▶) *T*_min_ = 0.703, *T*_max_ = 0.800
8803 measured reflections
4347 independent reflections
3848 reflections with *I* > 2σ(*I*)
*R*_int_ = 0.015

#### Refinement

*R*[*F* ^2^ > 2σ(*F* ^2^)] = 0.029
*wR*(*F* ^2^) = 0.076
*S* = 1.04
4347 reflections
331 parameters
2 restraints
H-atom parameters constrained
Δρ_max_ = 0.55 e Å^−3^
Δρ_min_ = −0.25 e Å^−3^

### 

Data collection: *SMART* (Bruker, 2007 ▶); cell refinement: *SAINT* (Bruker, 2007 ▶); data reduction: *SAINT*; program(s) used to solve structure: *SHELXS97* (Sheldrick, 2008 ▶); program(s) used to refine structure: *SHELXL97* (Sheldrick, 2008 ▶); molecular graphics: *SHELXTL* (Sheldrick, 2008 ▶); software used to prepare material for publication: *SHELXTL*.

## Supplementary Material

Crystal structure: contains datablocks global, I. DOI: 10.1107/S1600536808037331/lh2732sup1.cif

Structure factors: contains datablocks I. DOI: 10.1107/S1600536808037331/lh2732Isup2.hkl

Additional supplementary materials:  crystallographic information; 3D view; checkCIF report

## Figures and Tables

**Table:** 

Cu1—O1	1.9411 (15)
Cu1—N2	2.0207 (17)
Cu1—N1	2.0266 (17)
Cu1—N4	2.0471 (17)
Cu1—N3	2.1940 (18)

**Table:** 

O1—Cu1—N2	91.45 (7)
O1—Cu1—N1	167.87 (6)
N2—Cu1—N1	80.14 (7)
O1—Cu1—N4	91.03 (7)
N2—Cu1—N4	166.61 (7)
N1—Cu1—N4	95.20 (7)
O1—Cu1—N3	94.44 (7)
N2—Cu1—N3	115.38 (7)
N1—Cu1—N3	97.05 (7)
N4—Cu1—N3	77.52 (7)

**Table 2:** Hydrogen-bond geometry (Å, °)

*D*—H⋯*A*	*D*—H	H⋯*A*	*D*⋯*A*	*D*—H⋯*A*
C16—H16*A*⋯O5^i^	0.93	2.49	3.339 (3)	151
C12—H12*B*⋯O3	0.96	2.46	3.414 (3)	174
C8—H8*A*⋯O6^ii^	0.93	2.59	3.295 (3)	133
C7—H7*A*⋯O2^ii^	0.93	2.39	3.286 (3)	162
C4—H4*A*⋯O2^ii^	0.93	2.58	3.482 (3)	163
C2—H2*A*⋯O4	0.93	2.56	3.454 (3)	162
C1—H1*A*⋯O1	0.93	2.49	2.992 (3)	114

Symmetry codes: (i) 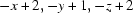; (ii) 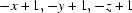.

**Fig. 1.**
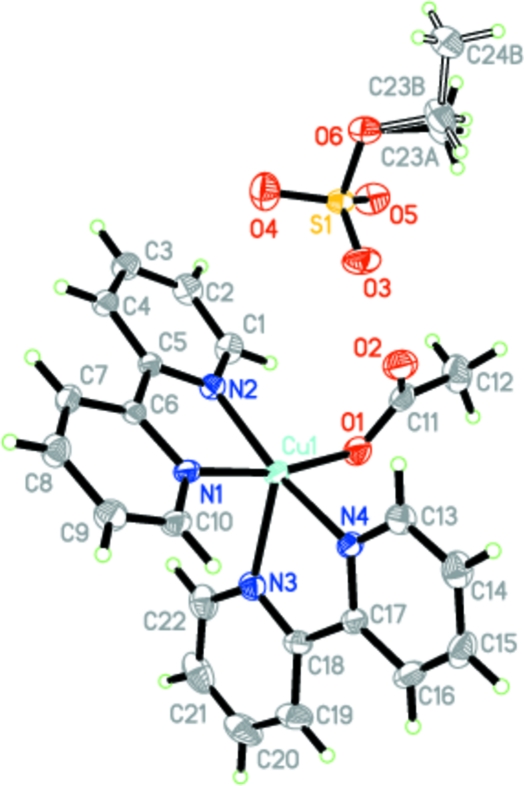
The molecular structure with displacement ellipsoids at the 30% probability level. The disorder is shown as open bonds.

**Fig. 2.**
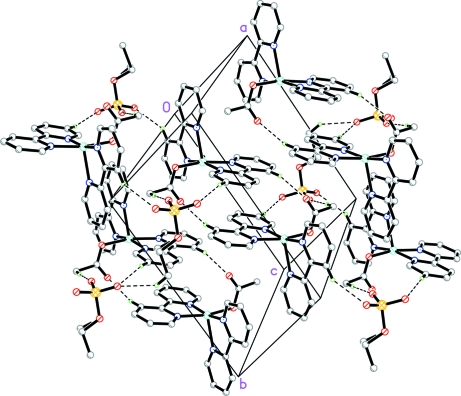
Part of the crystal structure showing hydrogen bonds as dashed lines. H atoms, except for those involved in hydrogen bonds, are not included.

## supplementary crystallographic information

### Comment

The field of supramolecular assembly and crystal engineering in which transition metal cationic centres are linked through anions *via* hydrogen-bonded supramolecular synthons is receiving growing attention (Yaghi *et al.*, 1998; Kitagawa *et al.*, 2004; Lu *et al.*, 2006). This work is driven by the elegant multi-dimensional architectures which can be fabricated by bringing together the rapidly maturing fields of hydrogen-bonded crystal engineering inorganic co-ordination polymer construction (Aakeröy *et al.*, 1998; Batten *et al.*, 1998). In the synthethis of the title compound,  methylsulfate and ethylsulfate are produced in two two stages (Blake *et al.*,  2000).

Herein, we report the synthesis and crystal structure of the title compound, (I), containing a discrete copper(II) complex cation and a disordered mixture of equal amounts of ethyl sulfate and methyl sulfate anions. The molecular structure of (I) is shown in Fig. 1. The Cu^II^ ion is chelated by two 2,2'-bipyridine ligands and is bonded to one oxygen of acetate moiety ion  forming a CuN_4_O distorted square-pyramidal coordination environment. In the crystal structure weak C-H···O hydrogen bonds link complex cations and sulfonate anions to form a three-dimensional network (Fig.2 and Table 2). Some crystal structures which are closely related to the title compound have already been studied (Blake *et al.*, 2000; Lopez-Sandoval *et al.*, 2004; Belokon *et al.*, 2002; Akrivos *et al.*, 1994).

### Experimental

Reagents and solvents used were of commercially available quality. To an aqueous solution (10 ml) of aminomethanesulfonic acid (0.11 g,1 mmol) and NaOH (0.04 g,1.0 mmol), Cu(CH_3_COO)_2_.H_2_O (0.20 g, 1.0 mmol) in methanol (10 ml) was added slowly. The solution was stirred for 30 min and then 2,2^'^-bipyridine (0.156 g, 1 mmol) in ethanol (10 ml) was added slowly. The mixture was refluxed overnight to give a green solution. After filtration, the solution was allowed to stand in air and after several days, green block-shaped crystal were collected in 20% yield. Analysis found: C 50.72, 30, H 4.22, N 10.16, S 5.72%; calculated for C_47_H_46_Cu_2_N_8_O_12_S_2_: C 50.90, H 4.16, N 10.12, S 5.78%.

### Refinement

H atoms were positioned geometrically and refined using a riding model, with C—H = 0.93–0.97 Å and with *U*_iso_(H) = 1.2*U*_eq_(C) or 1.5*U*_eq_(C) for methyl H atoms. From an initial solution irregular bond lengths, large displacement parameters in the C atoms of the anion and the presence of large peaks in difference Fourier maps which were close to the terminal (C_2_H_5_–) group, led us to suspect the presence of the disorder. The initially refined ratio of the site-occupany factors for the disorder components were eventually fixed at 0.5/0.5.

### Crystal data

**Table:**

[Cu(C_2_H_3_O_2_)(C_10_H_8_N_2_)_2_](C_2_H_5_O_4_S)_0.5_(CH_3_O_4_S)_0.5_	*Z* = 2
*M**_r_* = 553.06	*F*_000_ = 570
Triclinic, *P*1	*D*_x_ = 1.564 Mg m^−^^3^
Hall symbol: -P 1	Mo *K*α radiation λ = 0.71073 Å
*a* = 7.1314 (7) Å	Cell parameters from 4397 reflections
*b* = 13.1173 (13) Å	θ = 2.3–28.1º
*c* = 13.2783 (14) Å	µ = 1.07 mm^−^^1^
α = 91.875 (1)º	*T* = 291 (2) K
β = 104.673 (1)º	Block, green
γ = 101.162 (1)º	0.36 × 0.27 × 0.22 mm
*V* = 1174.5 (2) Å^3^	

### Data collection

**Table:**

Bruker SMART CCD area-detector diffractometer	4347 independent reflections
Radiation source: fine-focus sealed tube	3848 reflections with *I* > 2σ(*I*)
Monochromator: graphite	*R*_int_ = 0.015
*T* = 291(2) K	θ_max_ = 25.5º
φ and ω scans	θ_min_ = 2.3º
Absorption correction: multi-scan(SADABS; Sheldrick, 1996)	*h* = −8→8
*T*_min_ = 0.703, *T*_max_ = 0.800	*k* = −15→15
8803 measured reflections	*l* = −16→16

### Refinement

**Table:**

Refinement on *F*^2^	Secondary atom site location: difference Fourier map
Least-squares matrix: full	Hydrogen site location: inferred from neighbouring sites
*R*[*F*^2^ > 2σ(*F*^2^)] = 0.029	H-atom parameters constrained
*wR*(*F*^2^) = 0.076	*w* = 1/[σ^2^(*F*_o_^2^) + (0.0354*P*)^2^ + 0.5539*P*] where *P* = (*F*_o_^2^ + 2*F*_c_^2^)/3
*S* = 1.04	(Δ/σ)_max_ = 0.001
4347 reflections	Δρ_max_ = 0.55 e Å^−^^3^
331 parameters	Δρ_min_ = −0.25 e Å^−^^3^
2 restraints	Extinction correction: none
Primary atom site location: structure-invariant direct methods	

### Special details

**Table:**

Geometry. All e.s.d.'s (except the e.s.d. in the dihedral angle between two l.s. planes) are estimated using the full covariance matrix. The cell e.s.d.'s are taken into account individually in the estimation of e.s.d.'s in distances, angles and torsion angles; correlations between e.s.d.'s in cell parameters are only used when they are defined by crystal symmetry. An approximate (isotropic) treatment of cell e.s.d.'s is used for estimating e.s.d.'s involving l.s. planes.
Refinement. Refinement of *F*^2^ against ALL reflections. The weighted *R*-factor *wR* and goodness of fit *S* are based on *F*^2^, conventional *R*-factors *R* are based on *F*, with *F* set to zero for negative *F*^2^. The threshold expression of *F*^2^ > σ(*F*^2^) is used only for calculating *R*-factors(gt) *etc*. and is not relevant to the choice of reflections for refinement. *R*-factors based on *F*^2^ are statistically about twice as large as those based on *F*, and *R*- factors based on ALL data will be even larger.

### Fractional atomic coordinates and isotropic or equivalent isotropic displacement parameters (Å^2^)

**Table:**

	*x*	*y*	*z*	*U*_iso_*/*U*_eq_	Occ. (<1)
Cu1	0.69835 (4)	0.608241 (18)	0.760447 (18)	0.03168 (9)	
O1	0.8543 (2)	0.52120 (12)	0.84602 (12)	0.0433 (4)	
O2	0.9954 (3)	0.51379 (14)	0.71558 (14)	0.0550 (4)	
N1	0.5337 (2)	0.67731 (13)	0.64598 (13)	0.0317 (4)	
N2	0.4968 (2)	0.48175 (13)	0.68357 (13)	0.0323 (4)	
N3	0.6247 (3)	0.68159 (15)	0.89221 (14)	0.0383 (4)	
N4	0.9261 (2)	0.73567 (13)	0.80779 (13)	0.0326 (4)	
C1	0.4895 (3)	0.38259 (17)	0.70852 (18)	0.0415 (5)	
H1A	0.5772	0.3699	0.7694	0.050*	
C2	0.3570 (3)	0.29897 (18)	0.6473 (2)	0.0461 (6)	
H2A	0.3552	0.2313	0.6666	0.055*	
C3	0.2279 (3)	0.31788 (17)	0.55725 (19)	0.0430 (5)	
H3A	0.1371	0.2629	0.5149	0.052*	
C4	0.2337 (3)	0.41910 (16)	0.52997 (17)	0.0365 (5)	
H4A	0.1480	0.4329	0.4689	0.044*	
C5	0.3697 (3)	0.49981 (15)	0.59519 (15)	0.0289 (4)	
C6	0.3895 (3)	0.61088 (15)	0.57390 (15)	0.0284 (4)	
C7	0.2728 (3)	0.64571 (17)	0.48789 (16)	0.0362 (5)	
H7A	0.1754	0.5988	0.4389	0.043*	
C8	0.3031 (3)	0.75132 (18)	0.47579 (18)	0.0434 (5)	
H8A	0.2278	0.7761	0.4176	0.052*	
C9	0.4454 (4)	0.81974 (18)	0.55037 (19)	0.0469 (6)	
H9A	0.4651	0.8912	0.5443	0.056*	
C10	0.5578 (3)	0.78016 (16)	0.63401 (18)	0.0403 (5)	
H10A	0.6543	0.8263	0.6843	0.048*	
C11	0.9728 (3)	0.48732 (17)	0.80077 (19)	0.0424 (5)	
C12	1.0787 (4)	0.4086 (2)	0.8584 (3)	0.0645 (8)	
H12A	1.1963	0.4071	0.8362	0.097*	
H12B	0.9929	0.3408	0.8434	0.097*	
H12C	1.1144	0.4280	0.9322	0.097*	
C13	1.0729 (3)	0.75999 (18)	0.76051 (18)	0.0424 (5)	
H13A	1.0669	0.7195	0.7005	0.051*	
C14	1.2314 (4)	0.84204 (19)	0.7971 (2)	0.0516 (6)	
H14A	1.3283	0.8584	0.7613	0.062*	
C15	1.2432 (4)	0.89909 (19)	0.8875 (2)	0.0531 (7)	
H15A	1.3504	0.9541	0.9147	0.064*	
C16	1.0960 (4)	0.87503 (18)	0.93812 (19)	0.0467 (6)	
H16A	1.1037	0.9132	0.9999	0.056*	
C17	0.9355 (3)	0.79297 (16)	0.89594 (15)	0.0338 (5)	
C18	0.7654 (3)	0.76315 (16)	0.94229 (16)	0.0358 (5)	
C19	0.7519 (4)	0.8162 (2)	1.03148 (18)	0.0531 (6)	
H19A	0.8519	0.8722	1.0654	0.064*	
C20	0.5900 (5)	0.7851 (3)	1.0690 (2)	0.0658 (8)	
H20A	0.5784	0.8202	1.1284	0.079*	
C21	0.4449 (5)	0.7017 (3)	1.0183 (2)	0.0660 (8)	
H21A	0.3340	0.6793	1.0428	0.079*	
C22	0.4667 (4)	0.6515 (2)	0.9297 (2)	0.0532 (6)	
H22A	0.3684	0.5951	0.8951	0.064*	
S1	0.65876 (9)	0.06973 (4)	0.75424 (4)	0.04017 (14)	
O3	0.7495 (3)	0.17515 (13)	0.79359 (16)	0.0663 (5)	
O4	0.4498 (3)	0.05525 (16)	0.70285 (18)	0.0724 (6)	
O5	0.7039 (3)	−0.00602 (14)	0.82714 (14)	0.0622 (5)	
O6	0.7439 (3)	0.04364 (14)	0.65787 (13)	0.0530 (4)	
C23A	0.9552 (16)	0.054 (5)	0.690 (4)	0.066 (4)	0.50
H23A	1.0010	0.0371	0.6304	0.099*	0.50
H23B	0.9895	0.0082	0.7426	0.099*	0.50
H23C	1.0165	0.1250	0.7167	0.099*	0.50
C23B	0.9520 (17)	0.047 (5)	0.674 (4)	0.066 (4)	0.50
H23D	0.9971	0.0023	0.7275	0.079*	0.50
H23E	1.0274	0.1176	0.6951	0.079*	0.50
C24B	0.9788 (9)	0.0102 (6)	0.5741 (5)	0.0774 (19)	0.50
H24A	1.1174	0.0151	0.5804	0.116*	0.50
H24B	0.9268	0.0526	0.5207	0.116*	0.50
H24C	0.9097	−0.0610	0.5558	0.116*	0.50

### Atomic displacement parameters (Å^2^)

**Table:**

	*U*^11^	*U*^22^	*U*^33^	*U*^12^	*U*^13^	*U*^23^
Cu1	0.03401 (15)	0.02954 (14)	0.02825 (14)	0.00588 (10)	0.00340 (10)	−0.00086 (10)
O1	0.0449 (9)	0.0416 (9)	0.0391 (9)	0.0128 (7)	0.0004 (7)	0.0030 (7)
O2	0.0610 (11)	0.0528 (11)	0.0479 (10)	0.0116 (9)	0.0098 (9)	−0.0044 (8)
N1	0.0361 (9)	0.0272 (9)	0.0297 (9)	0.0068 (7)	0.0056 (7)	−0.0029 (7)
N2	0.0332 (9)	0.0299 (9)	0.0330 (9)	0.0052 (7)	0.0081 (7)	0.0034 (7)
N3	0.0376 (10)	0.0447 (11)	0.0345 (10)	0.0115 (8)	0.0108 (8)	0.0041 (8)
N4	0.0324 (9)	0.0318 (9)	0.0307 (9)	0.0053 (7)	0.0049 (7)	−0.0002 (7)
C1	0.0423 (12)	0.0370 (12)	0.0442 (13)	0.0056 (10)	0.0103 (10)	0.0123 (10)
C2	0.0457 (13)	0.0294 (12)	0.0638 (16)	0.0025 (10)	0.0192 (12)	0.0098 (11)
C3	0.0388 (12)	0.0316 (12)	0.0538 (14)	−0.0026 (9)	0.0121 (11)	−0.0040 (10)
C4	0.0331 (11)	0.0356 (11)	0.0376 (12)	0.0030 (9)	0.0074 (9)	−0.0022 (9)
C5	0.0261 (10)	0.0302 (10)	0.0311 (10)	0.0043 (8)	0.0104 (8)	0.0005 (8)
C6	0.0262 (10)	0.0290 (10)	0.0306 (10)	0.0052 (8)	0.0093 (8)	−0.0011 (8)
C7	0.0314 (11)	0.0385 (12)	0.0352 (11)	0.0056 (9)	0.0041 (9)	0.0013 (9)
C8	0.0429 (13)	0.0426 (13)	0.0423 (13)	0.0132 (10)	0.0030 (10)	0.0099 (10)
C9	0.0539 (14)	0.0297 (12)	0.0549 (15)	0.0116 (10)	0.0082 (12)	0.0057 (10)
C10	0.0444 (12)	0.0278 (11)	0.0433 (13)	0.0065 (9)	0.0037 (10)	−0.0039 (9)
C11	0.0365 (12)	0.0317 (11)	0.0480 (14)	0.0029 (9)	−0.0045 (10)	−0.0050 (10)
C12	0.0500 (15)	0.0452 (15)	0.095 (2)	0.0167 (12)	0.0066 (15)	0.0143 (15)
C13	0.0427 (13)	0.0403 (12)	0.0444 (13)	0.0061 (10)	0.0141 (10)	0.0037 (10)
C14	0.0409 (13)	0.0444 (14)	0.0699 (17)	0.0048 (11)	0.0175 (12)	0.0134 (12)
C15	0.0389 (13)	0.0372 (13)	0.0699 (18)	−0.0023 (10)	−0.0019 (12)	0.0053 (12)
C16	0.0533 (14)	0.0340 (12)	0.0420 (13)	0.0071 (10)	−0.0044 (11)	−0.0055 (10)
C17	0.0393 (11)	0.0297 (11)	0.0295 (10)	0.0118 (9)	0.0004 (9)	0.0029 (8)
C18	0.0480 (13)	0.0343 (11)	0.0267 (10)	0.0174 (10)	0.0059 (9)	0.0039 (8)
C19	0.0791 (18)	0.0499 (15)	0.0360 (13)	0.0268 (13)	0.0160 (13)	−0.0002 (11)
C20	0.092 (2)	0.081 (2)	0.0448 (15)	0.0477 (19)	0.0317 (16)	0.0100 (14)
C21	0.0636 (18)	0.101 (2)	0.0576 (17)	0.0440 (18)	0.0362 (15)	0.0336 (17)
C22	0.0437 (14)	0.0680 (18)	0.0526 (15)	0.0148 (12)	0.0176 (12)	0.0150 (13)
S1	0.0482 (3)	0.0304 (3)	0.0421 (3)	0.0057 (2)	0.0144 (3)	0.0001 (2)
O3	0.0808 (13)	0.0359 (10)	0.0769 (13)	−0.0036 (9)	0.0255 (11)	−0.0142 (9)
O4	0.0535 (11)	0.0638 (13)	0.0943 (16)	0.0101 (10)	0.0111 (11)	0.0104 (11)
O5	0.0844 (14)	0.0543 (11)	0.0475 (10)	0.0122 (10)	0.0173 (9)	0.0154 (9)
O6	0.0659 (11)	0.0516 (10)	0.0441 (9)	0.0147 (9)	0.0175 (8)	0.0019 (8)
C23A	0.0588 (18)	0.083 (7)	0.068 (10)	0.029 (2)	0.0290 (18)	0.013 (7)
C23B	0.0588 (18)	0.083 (7)	0.068 (10)	0.029 (2)	0.0290 (18)	0.013 (7)
C24B	0.064 (4)	0.094 (5)	0.080 (4)	0.021 (3)	0.028 (3)	−0.013 (4)

### Geometric parameters (Å, °)

**Table:**

Cu1—O1	1.9411 (15)	C12—H12B	0.9600
Cu1—N2	2.0207 (17)	C12—H12C	0.9600
Cu1—N1	2.0266 (17)	C13—C14	1.374 (3)
Cu1—N4	2.0471 (17)	C13—H13A	0.9300
Cu1—N3	2.1940 (18)	C14—C15	1.369 (4)
O1—C11	1.287 (3)	C14—H14A	0.9300
O2—C11	1.234 (3)	C15—C16	1.377 (4)
N1—C10	1.346 (3)	C15—H15A	0.9300
N1—C6	1.354 (2)	C16—C17	1.393 (3)
N2—C1	1.347 (3)	C16—H16A	0.9300
N2—C5	1.348 (3)	C17—C18	1.488 (3)
N3—C18	1.339 (3)	C18—C19	1.388 (3)
N3—C22	1.341 (3)	C19—C20	1.367 (4)
N4—C13	1.345 (3)	C19—H19A	0.9300
N4—C17	1.350 (3)	C20—C21	1.372 (4)
C1—C2	1.381 (3)	C20—H20A	0.9300
C1—H1A	0.9300	C21—C22	1.386 (4)
C2—C3	1.374 (3)	C21—H21A	0.9300
C2—H2A	0.9300	C22—H22A	0.9300
C3—C4	1.383 (3)	S1—O3	1.4279 (18)
C3—H3A	0.9300	S1—O5	1.4341 (18)
C4—C5	1.389 (3)	S1—O4	1.445 (2)
C4—H4A	0.9300	S1—O6	1.6026 (17)
C5—C6	1.480 (3)	O6—C23A	1.436 (7)
C6—C7	1.380 (3)	O6—C23B	1.438 (7)
C7—C8	1.381 (3)	C23A—H23A	0.9600
C7—H7A	0.9300	C23A—H23B	0.9600
C8—C9	1.377 (3)	C23A—H23C	0.9600
C8—H8A	0.9300	C23B—C24B	1.46 (5)
C9—C10	1.376 (3)	C23B—H23D	0.9700
C9—H9A	0.9300	C23B—H23E	0.9700
C10—H10A	0.9300	C24B—H24A	0.9600
C11—C12	1.511 (3)	C24B—H24B	0.9600
C12—H12A	0.9600	C24B—H24C	0.9600
			
O1—Cu1—N2	91.45 (7)	H12A—C12—H12B	109.5
O1—Cu1—N1	167.87 (6)	C11—C12—H12C	109.5
N2—Cu1—N1	80.14 (7)	H12A—C12—H12C	109.5
O1—Cu1—N4	91.03 (7)	H12B—C12—H12C	109.5
N2—Cu1—N4	166.61 (7)	N4—C13—C14	122.8 (2)
N1—Cu1—N4	95.20 (7)	N4—C13—H13A	118.6
O1—Cu1—N3	94.44 (7)	C14—C13—H13A	118.6
N2—Cu1—N3	115.38 (7)	C15—C14—C13	118.3 (2)
N1—Cu1—N3	97.05 (7)	C15—C14—H14A	120.8
N4—Cu1—N3	77.52 (7)	C13—C14—H14A	120.8
C11—O1—Cu1	112.76 (15)	C14—C15—C16	120.0 (2)
C10—N1—C6	118.32 (18)	C14—C15—H15A	120.0
C10—N1—Cu1	126.67 (14)	C16—C15—H15A	120.0
C6—N1—Cu1	114.99 (13)	C15—C16—C17	119.3 (2)
C1—N2—C5	118.53 (18)	C15—C16—H16A	120.3
C1—N2—Cu1	125.94 (15)	C17—C16—H16A	120.3
C5—N2—Cu1	115.32 (13)	N4—C17—C16	120.6 (2)
C18—N3—C22	118.5 (2)	N4—C17—C18	115.85 (18)
C18—N3—Cu1	113.03 (14)	C16—C17—C18	123.6 (2)
C22—N3—Cu1	128.37 (17)	N3—C18—C19	121.8 (2)
C13—N4—C17	118.92 (18)	N3—C18—C17	115.71 (18)
C13—N4—Cu1	123.50 (14)	C19—C18—C17	122.5 (2)
C17—N4—Cu1	117.38 (14)	C20—C19—C18	119.3 (3)
N2—C1—C2	122.6 (2)	C20—C19—H19A	120.3
N2—C1—H1A	118.7	C18—C19—H19A	120.3
C2—C1—H1A	118.7	C19—C20—C21	119.4 (3)
C3—C2—C1	118.6 (2)	C19—C20—H20A	120.3
C3—C2—H2A	120.7	C21—C20—H20A	120.3
C1—C2—H2A	120.7	C20—C21—C22	118.7 (3)
C2—C3—C4	119.7 (2)	C20—C21—H21A	120.6
C2—C3—H3A	120.2	C22—C21—H21A	120.6
C4—C3—H3A	120.2	N3—C22—C21	122.3 (3)
C3—C4—C5	118.9 (2)	N3—C22—H22A	118.9
C3—C4—H4A	120.5	C21—C22—H22A	118.9
C5—C4—H4A	120.5	O3—S1—O5	114.52 (12)
N2—C5—C4	121.65 (18)	O3—S1—O4	113.33 (13)
N2—C5—C6	114.68 (17)	O5—S1—O4	113.34 (12)
C4—C5—C6	123.66 (18)	O3—S1—O6	107.18 (11)
N1—C6—C7	121.76 (18)	O5—S1—O6	106.19 (11)
N1—C6—C5	114.55 (17)	O4—S1—O6	100.85 (12)
C7—C6—C5	123.69 (18)	C23A—O6—S1	111.6 (19)
C6—C7—C8	119.0 (2)	C23B—O6—S1	120.4 (18)
C6—C7—H7A	120.5	O6—C23A—H23A	109.5
C8—C7—H7A	120.5	O6—C23A—H23B	109.5
C9—C8—C7	119.6 (2)	O6—C23A—H23C	109.5
C9—C8—H8A	120.2	O6—C23B—C24B	107 (2)
C7—C8—H8A	120.2	O6—C23B—H23D	110.3
C10—C9—C8	118.6 (2)	C24B—C23B—H23D	110.3
C10—C9—H9A	120.7	O6—C23B—H23E	110.3
C8—C9—H9A	120.7	C24B—C23B—H23E	110.3
N1—C10—C9	122.6 (2)	H23D—C23B—H23E	108.6
N1—C10—H10A	118.7	C23B—C24B—H24A	109.5
C9—C10—H10A	118.7	C23B—C24B—H24B	109.5
O2—C11—O1	123.5 (2)	H24A—C24B—H24B	109.5
O2—C11—C12	121.8 (2)	C23B—C24B—H24C	109.5
O1—C11—C12	114.7 (2)	H24A—C24B—H24C	109.5
C11—C12—H12A	109.5	H24B—C24B—H24C	109.5
C11—C12—H12B	109.5		
			
N2—Cu1—O1—C11	83.73 (15)	C10—N1—C6—C5	−178.14 (18)
N1—Cu1—O1—C11	37.9 (4)	Cu1—N1—C6—C5	3.6 (2)
N4—Cu1—O1—C11	−83.11 (15)	N2—C5—C6—N1	0.6 (2)
N3—Cu1—O1—C11	−160.68 (15)	C4—C5—C6—N1	−178.70 (18)
O1—Cu1—N1—C10	−136.1 (3)	N2—C5—C6—C7	−179.65 (18)
N2—Cu1—N1—C10	177.24 (19)	C4—C5—C6—C7	1.0 (3)
N4—Cu1—N1—C10	−15.43 (18)	N1—C6—C7—C8	−0.6 (3)
N3—Cu1—N1—C10	62.60 (18)	C5—C6—C7—C8	179.67 (19)
O1—Cu1—N1—C6	42.0 (4)	C6—C7—C8—C9	−1.4 (3)
N2—Cu1—N1—C6	−4.66 (13)	C7—C8—C9—C10	1.8 (4)
N4—Cu1—N1—C6	162.67 (14)	C6—N1—C10—C9	−1.7 (3)
N3—Cu1—N1—C6	−119.29 (14)	Cu1—N1—C10—C9	176.35 (17)
O1—Cu1—N2—C1	8.46 (18)	C8—C9—C10—N1	−0.2 (4)
N1—Cu1—N2—C1	179.67 (18)	Cu1—O1—C11—O2	5.2 (3)
N4—Cu1—N2—C1	109.1 (3)	Cu1—O1—C11—C12	−172.77 (16)
N3—Cu1—N2—C1	−87.15 (18)	C17—N4—C13—C14	−1.1 (3)
O1—Cu1—N2—C5	−166.17 (14)	Cu1—N4—C13—C14	−175.82 (17)
N1—Cu1—N2—C5	5.04 (14)	N4—C13—C14—C15	2.3 (4)
N4—Cu1—N2—C5	−65.5 (3)	C13—C14—C15—C16	−1.4 (4)
N3—Cu1—N2—C5	98.22 (14)	C14—C15—C16—C17	−0.5 (4)
O1—Cu1—N3—C18	84.49 (15)	C13—N4—C17—C16	−0.9 (3)
N2—Cu1—N3—C18	178.22 (13)	Cu1—N4—C17—C16	174.12 (15)
N1—Cu1—N3—C18	−99.38 (14)	C13—N4—C17—C18	178.51 (18)
N4—Cu1—N3—C18	−5.59 (14)	Cu1—N4—C17—C18	−6.4 (2)
O1—Cu1—N3—C22	−91.6 (2)	C15—C16—C17—N4	1.7 (3)
N2—Cu1—N3—C22	2.1 (2)	C15—C16—C17—C18	−177.7 (2)
N1—Cu1—N3—C22	84.5 (2)	C22—N3—C18—C19	0.4 (3)
N4—Cu1—N3—C22	178.3 (2)	Cu1—N3—C18—C19	−176.14 (17)
O1—Cu1—N4—C13	86.96 (17)	C22—N3—C18—C17	−179.43 (19)
N2—Cu1—N4—C13	−13.7 (4)	Cu1—N3—C18—C17	4.0 (2)
N1—Cu1—N4—C13	−82.63 (17)	N4—C17—C18—N3	1.3 (3)
N3—Cu1—N4—C13	−178.72 (18)	C16—C17—C18—N3	−179.33 (19)
O1—Cu1—N4—C17	−87.84 (15)	N4—C17—C18—C19	−178.58 (19)
N2—Cu1—N4—C17	171.5 (2)	C16—C17—C18—C19	0.8 (3)
N1—Cu1—N4—C17	102.57 (15)	N3—C18—C19—C20	−0.6 (4)
N3—Cu1—N4—C17	6.48 (14)	C17—C18—C19—C20	179.3 (2)
C5—N2—C1—C2	−0.1 (3)	C18—C19—C20—C21	0.5 (4)
Cu1—N2—C1—C2	−174.60 (17)	C19—C20—C21—C22	−0.3 (4)
N2—C1—C2—C3	0.2 (4)	C18—N3—C22—C21	−0.2 (4)
C1—C2—C3—C4	0.2 (3)	Cu1—N3—C22—C21	175.77 (18)
C2—C3—C4—C5	−0.6 (3)	C20—C21—C22—N3	0.1 (4)
C1—N2—C5—C4	−0.3 (3)	O3—S1—O6—C23A	60 (3)
Cu1—N2—C5—C4	174.78 (15)	O5—S1—O6—C23A	−62 (3)
C1—N2—C5—C6	−179.62 (17)	O4—S1—O6—C23A	179 (3)
Cu1—N2—C5—C6	−4.6 (2)	O3—S1—O6—C23B	62 (3)
C3—C4—C5—N2	0.6 (3)	O5—S1—O6—C23B	−61 (3)
C3—C4—C5—C6	179.90 (19)	O4—S1—O6—C23B	−179 (3)
C10—N1—C6—C7	2.1 (3)	S1—O6—C23B—C24B	175 (2)
Cu1—N1—C6—C7	−176.14 (15)		

### Hydrogen-bond geometry (Å, °)

**Table:**

*D*—H···*A*	*D*—H	H···*A*	*D*···*A*	*D*—H···*A*
C16—H16A···O5^i^	0.93	2.49	3.339 (3)	151
C12—H12B···O3	0.96	2.46	3.414 (3)	174
C8—H8A···O6^ii^	0.93	2.59	3.295 (3)	133
C7—H7A···O2^ii^	0.93	2.39	3.286 (3)	162
C4—H4A···O2^ii^	0.93	2.58	3.482 (3)	163
C2—H2A···O4	0.93	2.56	3.454 (3)	162
C1—H1A···O1	0.93	2.49	2.992 (3)	114

Symmetry codes: (i) −*x*+2, −*y*+1, −*z*+2; (ii) −*x*+1, −*y*+1, −*z*+1.
